# Heart Team - the Reality in Some Groups

**DOI:** 10.21470/1678-9741-2019-0023

**Published:** 2019

**Authors:** Eduardo Augusto Victor Rocha

**Affiliations:** 1 Department of Cardiovascular Surgery, Faculdade da Saúde e Ecologia Humana (FASEH), Belo Horizonte, BH, Brazil.; 2 Department of Cardiovascular Surgery, Hospital Lifecenter, Belo Horizonte, BH, Brazil.

In the last issue 33.6, we saw Yadava OP^[1]^ talking about the reality of the Heart Team in developing countries. Now, I would like to discuss a little about the Heart Team in Brazil.

The Heart Team is a class I indication as the best approach for cardiac patients. The Heart Team has brought more patients to be treated by the surgical groups and, therefore, doesn't get much resistance from the surgeons. I see, even today, a great difficulty in having discussions in true Heart Teams. Oftentimes there are discussions from which surgeons are excluded. However, many colleagues, both clinical and hemodynamicist, argue that the group is a way to withdraw the autonomy of those doctors. The physician should be the moderating power; they play a key role in the Heart Team and therefore must be well prepared and have a strong scientific spirit. They cannot be biased and for this they must believe that the Heart Team should be called on even when their opinion is contradicted. There is no adequate Heart Team where there is vanity and little openness to the discussion. As physicians, we are people who habitually get used to overcoming personal challenges, we study continuously, but we find it difficult to be confronted, even with scientific arguments. To be effective in the treatment of our patients, we must, first of all, be humble. This is why we have two ears and one mouth: we need to listen more and only say as a part of three parts that form the Heart Team. We need to reprogram our minds to this new reality where knowledge is not restricted to our personal islands. A man is not an island.
